# Lasing optical cavities based on macroscopic scattering elements

**DOI:** 10.1038/srep40141

**Published:** 2017-01-10

**Authors:** Antonio Consoli, Cefe López

**Affiliations:** 1 Instituto de Ciencia de Materiales de Madrid, Consejo Superior de Investigaciones Científicas, Calle Sor Juana Ines de la Cruz 3, 28049 Madrid, Spain

## Abstract

Two major elements are required in a laser device: light confinement and light amplification. Light confinement is obtained in optical cavities by employing a pair of mirrors or by periodic spatial modulation of the refractive index as in photonic crystals and Bragg gratings. In random lasers, randomly placed nanoparticles embedded in the active material provide distributed optical feedback for lasing action. Recently, we demonstrated a novel architecture in which scattering nanoparticles and active element are spatially separated and random lasing is observed. Here we show that this approach can be extended to scattering media with macroscopic size, namely, a pair of sand grains, which act as feedback elements and output couplers, resulting in lasing emission. We demonstrate that the number of lasing modes depends on the surface roughness of the sand grains in use which affect the coherent feedback and thus the emission spectrum. Our findings offer a new perspective of material science and photonic structures, facilitating a novel and simple approach for the realization of new photonics devices based on natural scattering materials.

Scattering feedback for lasing action is obtained in random lasers (RLs) through random spatial distribution of scattering centers into an active element^1^,^2^. Seminal work on this idea appeared few years after the realization of the first laser^3^,^4^. Specifically, Ambartsumyan and colleagues proposed a “non-resonant” cavity in which one mirror of a classical Fabry-Perot cavity was substituted for a scattering element^5^. They obtained the emission line of the pumped material with no specific spectral signature due to any coherent feedback. This topic was almost abandoned for about 30 years until lasing emission was demonstrated from a colloidal solution^6^ and narrow optical resonances were obtained from semiconductor powders^7^. Since then, a rich variety of materials has been employed in RLs, e.g. powders of active materials^8^, dye doped biological tissue^9^, liquid crystals^10^, optical fibres^11^, and polymers^12^.

Applications of RLs range a wide span of fields, e.g. free laser based imaging systems^13^, optical tagging^14^, sensing at nanoscale^15^ and optical information processing^16^.

Random lasing has been divided in two main operational regimes: the so-called “resonant” and “non-resonant” emissions, associated to a multi peak spectrum with randomly placed narrow frequency modes and to a single peaked spectrum with few nanometer width, respectively^13^. Only recently, this behavior has been understood as a multimode emission in both cases, with the “non-resonant” regime consisting of a large number of modes mutually coupled together, resulting in a broad single-peaked spectrum^17^,^18^,^19^.

Generally, the term RL refers to a distributed architecture, in which scattering nanoparticles and gain material share the same space. Recently, we proposed a RL device architecture with spatially localized feedback, in which the gain material is placed between two large ensembles of randomly placed scattering nanoparticles, acting as feedback and output coupling elements^20^. Lasing action in such structure is understood as due to the feedback in amplitude and phase from scattering regions: narrow resonances are allowed to exist at spectral positions where a closed loop is established in the entire structure with an integer number of wavelengths. This corresponds to lasing modes randomly placed in frequency, due to the spectral phase response of the scattering regions, and with different thresholds and slopes, due to the frequency dependent losses resulting from the spectral amplitude response of the scattering regions. We observed the transition from the “resonant” to “non-resonant” operation regimes^21^ in a RL with spatially localized feedback in which, given the scattering material, the size of the pumped area is changed, promoting mutual interaction and strong coupling between lasing modes.

In conceiving the present work, we asked ourselves to which extent two scattering elements can support coherent lasing emission, which characteristics are relevant and how they affect the lasing spectral signature. We chose a simple macroscopic natural material, raw sand, and we built lasing devices using pairs of grains of three qualitatively different types of sand.

We selected three kinds of sand for which surface roughness constituted the essential difference. This magnitude has been quantified on 250 mg sand samples by specific surface area (SSA) measurements with Brunauer-Emmett-Teller (BET) technique. Grains from each type were observed with the scanning electron microscope (SEM) and their chemical composition obtained through energy-dispersive X-ray spectroscopy (EDX).

## Results

Devices are prepared by placing two grains of each sand sample on a glass substrate at a distance of about 3 mm and then cast a liquid drop of a dye-doped polymer blend, so that a solid film is formed after slowly heating the substrate. The polymer blend consists of a CTMA-DNA complex^22^ doped with DCM dye^12^ in ethanol.

**Figure 1:**
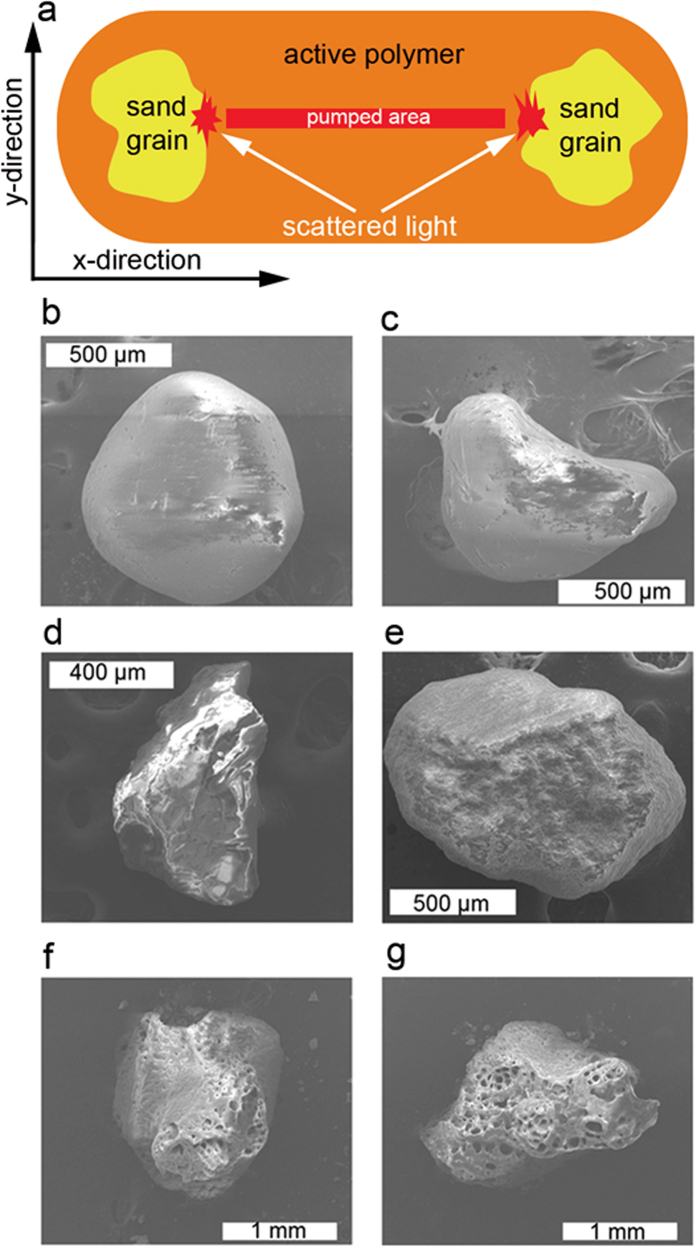
Optical cavities based on two grains of sand. (**a**) Schematic sketch of the experiments. (**b,c**) Sand grains used in device #1. (**d,e**) Sand grains used in device #2. (**f,g**) Sand grains used in device #3.

The obtained devices are optically pumped with an optical set-up based on an intensity-only spatial light modulator. This allows shaping the pump beam cross section so that a rectangular area placed between the two sand grains is pumped. In Fig. 1a, a schematic view of the experiment is given. Scattered light from the sand grains is collected with an optical setup, fed to a fiber and send to the spectrometer. Image acquisition is performed by imaging the sample onto a CCD camera. More experimental details about sample preparation and experimental set-up are given in Methods section.

Device #1 is obtained with grains of sand collected at Mission beach in San Diego, U. S. This sand consists of small stones of SiO_2_, showing a smooth surface and a small SSA = 0.76 m^2^/g. In Fig. 1b and 1c, the two grains used in the device are shown.

Device #2 is obtained with grains of sand collected in the Sahara desert in an area close to the city of Ouarzazate (Morocco). Sand composition shows a large percentage of SiO_2_ with some amounts of metals, mostly iron and aluminum, and microscope images show grains with multi-faceted surfaces. In Fig. 1d,e, two typical grains used in the device are shown. From BET measurements we obtained SSA = 1.38 m^2^/g.

Device #3 is obtained with grains of sand collected at Espalmador beach in Formentera (Spain). It consists of pinkish, large grains with irregular shape and porous surfaces. In Fig. 1f,g, the two grains used in the device are shown. Complex textural patterns recalling biological structures and a high content of calcium carbonate suggest origin from sea shells. For this sand sample we obtained SSA = 2.03 m^2^/g, corresponding to the highest value of the three samples under study.

All experiments are performed with a pumped area with horizontal length, along the x-direction in Fig. 1a, of 2.8 mm and vertical width, corresponding to the y-direction in Fig. 1a, of 50 μm.

**Figure 2:**
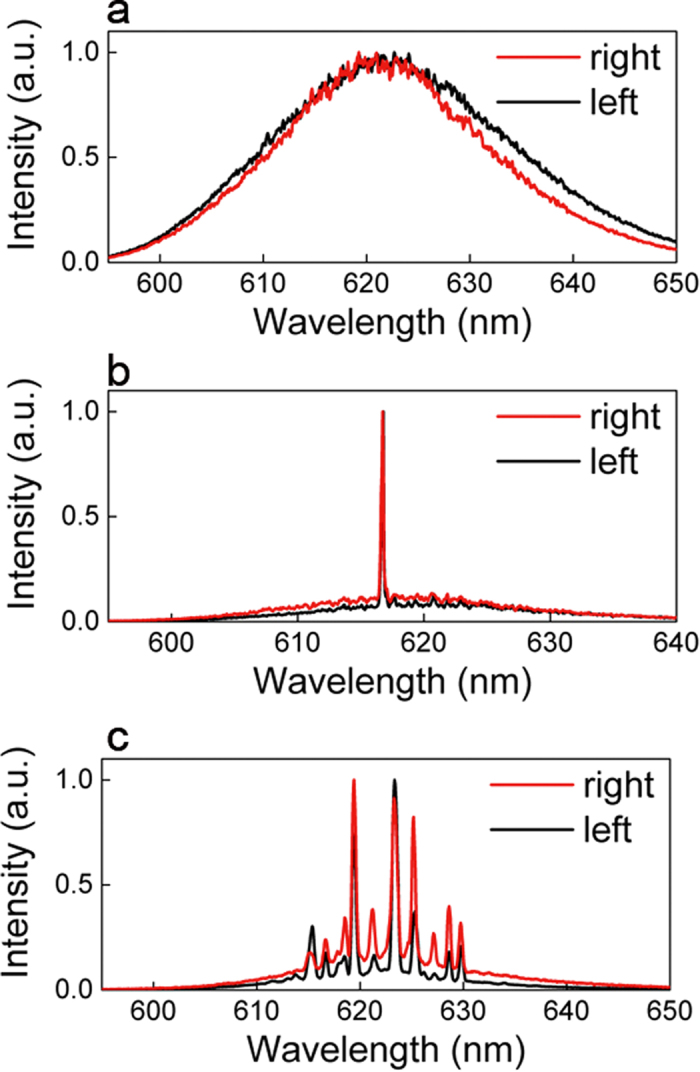
Emission spectra from the three sand lasers considered. Spectra collected from right (red line) and left (black line) sand grains of device #1, (**a**) device #2, (**b**) and device #3, (**c**). The pump energy is E_P_ = 53 pJ/μm^2^ in all experiments.

Representative emission spectra collected from the scattering points at right and left grains of each device are shown in Fig. 2. The pump energy is E_P_ = 53 pJ/μm^2^ in all experiments.

From device #1, we observe that typical spectra show a broad emission peak, with full width at half maximum (FWHM) of about 20 nm, corresponding to the amplified spontaneous emission (ASE) of the gain material. In this case, no lasing action is taking place and the two grains of sand are simply scattering off the plane the ASE flux exiting the pumped stripe.

The collected CCD image of the device shows two bright spots from left and right grain, see Supplementary Fig. 1a and 1b, respectively. The emission spectrum and the total emitted intensity as a function of E_P_ are shown in Supplementary Fig. 1c and 1d, respectively.

A typical lasing emission spectrum from device #2 shows a sharp peak with a resolution limited FWHM = 0.4 nm at 617.8 nm, see Fig. 2b. In this case, the back-scattered radiation from the grains re-enters the active medium and closes a feedback loop, resulting in lasing action^20^. The Q-factor of this optical cavity, calculated as Q = ν_0_/δν, where ν_0_ is the central frequency and δν is the linewidth, corresponds to Q = 1545. CCD images from left and right grains at the scattering points show a few local maxima and minima of intensity, due to spatial interference from back-scattered radiation, see Supplementary Fig. 2a and 2b. For a given position of the pumping stripe the spectral position of the observed peak is constant with pump energy and lasing threshold occurs at 25 pJ/μm^2^, as shown in Supplementary Fig. 2c and 2d, respectively.

Multi-mode emission is typically observed from device #3, with the same excited modes shining off from right and left grains, as shown in Fig. 2c where representative emission spectra are show. For this particular case the spectrum consists of 10 modes with no clear periodicity in frequency and a resolution limited FWHM for each mode. As for device #2, a feedback loop is established and lasing action takes place. Images from the scattering points at left and right grains are shown in Supplementary Fig. 3a and 3b, respectively. We observe a random pattern of intensity with several intensity hotspots, suggesting that spatial interference is taking place leading to the formation of the mode inside the sand grain. Emission spectra and total emitted intensity as a function of the pump energy are shown in Supplementary Fig. 3c and 3d. Modes start lasing at about 20 pJ/μm^2^, they maintain their frequency positions with pump and show different thresholds and slopes.

The theoretical framework in which the presented results are understood has been described in a previous work^20^, in which a similar system, consisting of an active material placed between two scattering elements formed by random distributions of titanium dioxide nanoparticles, was studied.

Each scattering element has been modeled with a complex spectral response with arbitrary amplitude and phase spectral profiles and introduced into the round trip condition for lasing^23^ as equivalent “mirror” of a Fabry-Perot cavity. At each frequency, the round trip equation has been numerically evaluated and modes are allowed to exist if an integer number of wavelengths satisfies the phase condition.

At variance with a classical mirror-based Fabry-Perot cavity, no optical feedback might be expected if two diffusive elements were used instead of mirrors. In such a device, no lasing action would take place due to the lack of optical feedback, with the output consisting of the diffused ASE which exits the pumped area. This is the behavior observed in device #1. However, the presence of narrow modes, as observed in device #2 and #3, is a clear clue to random lasing action and coherent optical feedback that we study as function of the SSA of the sand grains in use.

From the spectra shown in Fig. 2, we observe that, depending on the sand grains in use, different cases can show up: i) no lasing modes are allowed in the gain window, as for device #1 in Fig. 2a, ii) only a single mode exists and lase, as for device #2 in Fig. 2b, and (iii) the emission spectrum consists of several modes, as for device #3 in Fig. 2c.

According to our theoretical model, this corresponds to having different phase response for the three devices, as their scattering elements are characterized by different surface roughness and porosity, which in turn leads to no lasing action, single mode or multimode operation.

**Figure 3:**
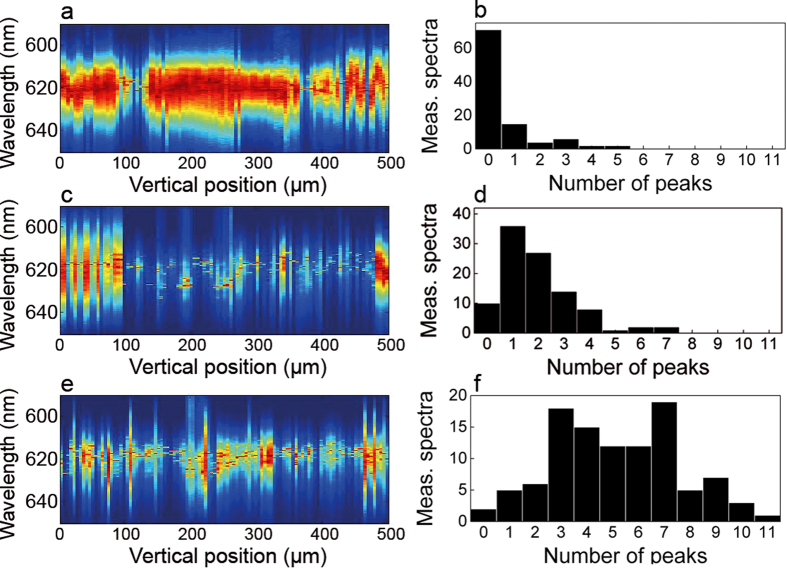
Emission as a function of the pump stripe vertical position. Measured spectra as a function of the pump vertical position and distribution of the number of detected peaks for device #1, (**a,b**) for device #2, (**c,d**) and for device #3, (**e,f**).

In Fig. 2, we reported the most representative cases obtained with each of our devices. In order to address how the local roughness of the sand grains affect lasing emission, we performed a statistical study by taking a set of 100 spectral measurements for each device by varying the pump stripe position along the y-direction with a 5 μm increment step and constant E_P_ = 53 pJ/μm^2^. A simple algorithm for peak detection and counting is numerically implemented, obtaining the statistical distribution of the number of detected peaks for each device. Results are shown in Fig. 3.

From all devices, we obtain ASE, single mode and multi-mode lasing emission. However, the distribution of the number of detected peaks varies qualitatively between devices. From device #1, ASE spectrum is observed in most of the cases and laser emission is rarely detected, see Fig. 3a and b. From device #2, see Fig. 3c and d, very few cases correspond to ASE, and most of the measurements show single mode spectra, where the maximum of the distribution is found. Spectra collected from device #3 consist mostly of multi-mode lasing emission, with 3 to 7 lasing modes, up to a maximum of 11 detected peaks.

## Discussion

These results are understood in the theoretical framework of RLs with spatially localized feedback^20^,^21^. Here, we demonstrate that the surface roughness and porosity of the scattering elements in use is crucial in determining the lasing regime leading to no lasing action, single mode or multimode emission. In this work, we used a pair of sand grains to demonstrate this concept. On a given pair of grains, all those different behaviors are observed, as the surface roughness, and thus the phase matching condition for lasing, can change along the grain extent.

Compared to seminal work on “non-resonant” lasers^4^,^5^, we proposed here a different approach based on a laser device consisting of an active material placed between two scattering elements altogether dispensing with mirrors. We investigate the emission characteristics as a function of the morphology, and consequent scattering properties, of the materials in use and, at variance with previously reported results^4^,^5^, we obtained ASE, single mode and multimode emissions, from devices #1, #2 and #3, respectively.

In the three devices considered, we observed a clear statistical behavior in which lasing action is usually inhibited in scattering elements with smooth surfaces because they are too similar to lossy cavities while rough and porous surfaces sustain single- and multi-mode emission.

In our vision, the relevance of the presented results is to break the preconception concerning the need of reflective surface for obtaining optical cavities and narrow lasing resonances. In principle, any scattering material could be used as feedback element (provided absorption is contained), resulting in omnidirectional laser light emission. This new kind of device would find use in a wide range of energy efficient practical applications in which diffuse light is required and open the way to new research scenarios in which coherent light amplification and scattering phenomena coexist. And that without the need for highly sophisticated materials and methods but merely using virtually inexhaustible resources.

## Methods

### Sample fabrication

The active element is a bio polymeric film obtained from a liquid solution of deoxyribonucleic acid (DNA) and cetyltrimethyl ammonium (CTMA) doped with 4-(Dicyanomethylene)-2-methyl-6-(4-dimethylaminostyryl)-4H-pyran (DCM) dye. The cationic surfactant CTMA is used to make the DNA soluble in organic solvents and then easily mixable with the DCM dye solution.

The DNA-CTMA complex is prepared by magnetic stirring for 2 hours an aqueous solution of DNA sodium salt (200 mL, 0.5 vol.%, Sigma-Aldrich D1626) and CTMA chloride (200 mL, 25 vol.% in water, Sigma-Aldrich 292737). The DNA-CTMA liquid complex is then precipitated for 5 hours, collected by centrifugation at 9000 rpm and finally dried at 70 °C for 12 hours. The result is a 1 gram spongy solid disc with 2 cm diameter and 2 mm thickness.

The DNA-CTMA complex is disolved (200 mg, 4 vol.%) in ethanol and mixed with 1.4 mL of the DCM dye solution (0.5 vol.% in equal parts of ethanol and chloroform). The resulting blend is magnetically stirred for 5 hours.

The lasing devices are fabricated by manually placing the two grains of sand onto a glass substrate at a distance of 3 mm. A small drop (10 μL) of the dye doped blend is then cast onto the substrate, covering a circular area of about 4 mm diameter, in which the sand grains are included. The sample is then dried with a 60 W lamp for 15 minutes.

### Experimental set-up

**Figure 4:**
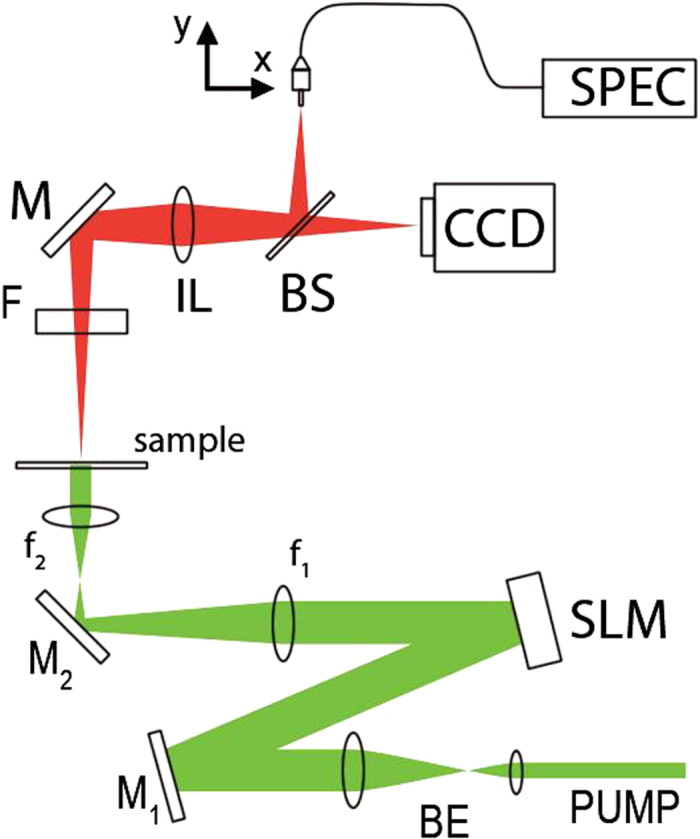
Experimental set-up. Details are given in the text.

The experimental set-up is shown in Fig. 4. The optical pump is a frequency doubled Nd:YAG Q-switched laser (Litron NanoT250) delivering 10 ns pulses at 532 nm with 10 Hz repetition rate. The pump beam passes through a beam expander (BE) in order to uniformly illuminate a spatial light modulator (SLM) operating in amplitude. Two polarization dichroic mirrors, placed before (M1) and after (M2) the SLM and oriented with orthogonal polarization axes, allow the modulation of the pump flux on the sample. By imposing a variable gray level to the SLM pixels, the incident polarization is rotated up to a maximum of 90° (white pixels) and the light intensity on the sample after M2 is finely tuned. The beam size is reduced (×0.15) after the SLM with a pair of convex lenses (f_1_, f_2_).

Residual pump after the sample is suppressed with a long pass edge filter (F) and the sample is imaged onto a CCD camera and onto the plane where the fiber end is placed with an imaging lens (IL) and a beam splitter (BS). The fiber end (105 μm diameter) collects light from the sample and sends it to the spectrometer (SPEC, Andor Shamrock 303). Two computer controlled translation stages allow the vertical and horizontal displacement of the fiber end onto the sample image.

## Additional Information

**How to cite this article**: Consoli, A. and López, C. Lasing optical cavities based on macroscopic scattering elements. *Sci. Rep.* **7**, 40141; doi: 10.1038/srep40141 (2017).

**Publisher's note:** Springer Nature remains neutral with regard to jurisdictional claims in published maps and institutional affiliations.

## Supplementary Material

Supplementary Information
