# Supplementary material for: Lasing optical cavities based on macroscopic scattering elements
Source: Sci Rep. 2017 Jan 10;7:40141. doi: 10.1038/srep40141 (PMC5223191; doi:10.1038/srep40141)
Supplement: Supplementary Information [file srep40141-s1.pdf]

Lasing optical cavities based on macroscopic scattering elements

Antonio Consoli* and Cefe López

Instituto de Ciencia de Materiales de Madrid, Consejo Superior de Investigaciones Científicas, Calle Sor Juana Ines de la Cruz 3, 28049 Madrid, Spain

* antonio.consoli@csic.es

Supplementary Information

Device Characterization

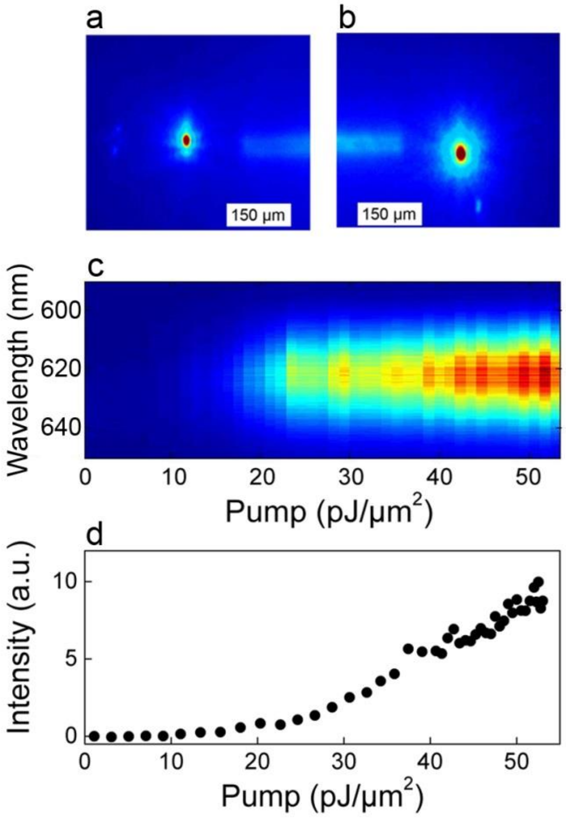

Supplementary Fig. 1. Experimental results from device #1. a, image detected from left grain for E_P = 53 pJ/µm^2. b, image detected from right grain for E_P = 53 pJ/µm^2.c, Measured spectra as a function of the pumping energy, d, Total emission intensity as a function of pump energy.

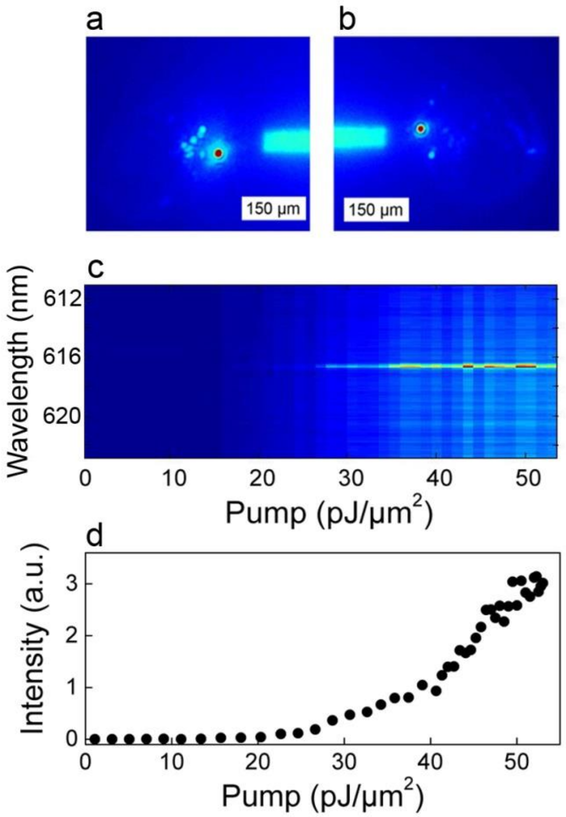

Supplementary Fig. 2. Experimental results from device #2. a, image detected from left grain for EP = 53 pJ/µm2. b, image detected from right grain for EP = 53 pJ/µm2.c, Measured spectra as a function of the pumping energy, d, Total emission intensity as a function of pump energy.

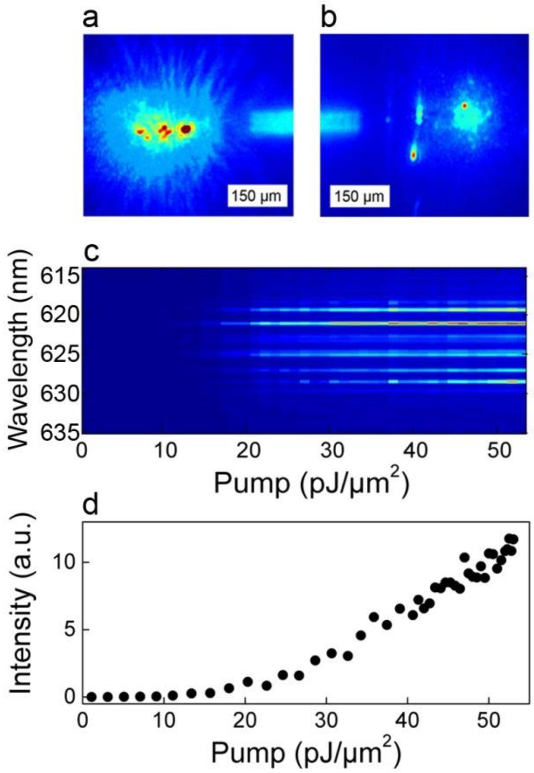

Supplementary Fig. 3. Experimental results from device #3. a, image detected from left grain for E_P = 53 pJ/µm^2. b, image detected from right grain for E_P = 53 pJ/µm^2.c, Measured spectra as a function of the pumping energy, d, Total emission intensity as a function of pump energy.
